# Knowledge and Awareness About Food Allergy Among Mothers With Allergic Children in the Aseer Region, Saudi Arabia

**DOI:** 10.7759/cureus.43801

**Published:** 2023-08-20

**Authors:** Zeinh H Fardan, Mohammed Abdullah Aoun Alshahrani, Reem T Alalyani, Arwa E Alshahrani, Renad M Alshehri, Nawaf Saleh M Alshamrani, Fatimah Obaid M Aldabali, Norah Saaed A Alqahtani, Khalid Siraj S Altalhiyyah, Mahdi Muhammad M Alqahtani

**Affiliations:** 1 Pediatrics, King Khalid University, Abha, SAU; 2 Medicine and Surgery, King Khalid University, Abha, SAU; 3 Pediatrics, Abha Maternity and Children Hospital, Abha, SAU; 4 Medicine and Surgery, Umm Al-Qura University, Al Qunfudhah, SAU

**Keywords:** allergies, awareness, knowledge, allergic children, mothers, food allergies

## Abstract

Introduction

Food allergies have become a significant health concern worldwide, affecting individuals of all age groups. It is particularly challenging for parents who have children diagnosed with food allergies, as they bear the responsibility of managing their child's condition and ensuring their safety. This study aimed to assess the knowledge and awareness about food allergies among mothers with allergic children in the Aseer region, Saudi Arabia.

Methodology

A cross-sectional study design was employed, and data were collected through a structured questionnaire administered to 400 mothers. The participants were selected through convenience sampling. Descriptive statistics, including frequencies and percentages, were used to summarize the demographic characteristics, knowledge and awareness levels, prevalence of allergenic foods, manifestations of food allergies, and factors influencing food allergies. Chi-squared tests were conducted to assess associations between variables.

Results

The study revealed a balanced representation across different age groups among the surveyed mothers, with the highest proportion falling in the 31-40 years range. A significant proportion of mothers had a university education, indicating a relatively higher level of education among the participants. The majority of mothers were employed in governmental positions, followed by housewives and those working in the private sector. Regarding knowledge and awareness, a substantial percentage of mothers correctly identified key aspects of food allergies. However, knowledge gaps were observed, particularly in understanding the hereditary nature. The prevalence of allergenic foods varied, with seafood (fish, shrimp, and tuna) emerging as the most commonly reported allergenic foods, followed by eggs, milk, wheat, chocolate, fruits, nuts, and other food types. Manifestations associated with food allergies were reported, including skin, respiratory, eye, nasal, gastrointestinal, and other symptoms. The associations between different feeding methods and the occurrence of medically diagnosed food allergies were found to be non-significant. However, having other children with food allergies showed a highly significant association with the occurrence of food allergies in the current child. The age of introducing solid food did not demonstrate a significant association with the occurrence of food allergies.

Conclusion

This study provides insights into the knowledge and awareness about food allergies among mothers with allergic children in the Aseer region, Saudi Arabia. While mothers demonstrated a reasonable understanding of food allergies, knowledge gaps were identified, particularly regarding the hereditary nature. The prevalence of allergenic foods aligns with previous studies, although variations across populations should be considered. The manifestations reported by the participants corroborate known allergic reactions, necessitating further analysis.

## Introduction

Food allergies have become a significant health concern worldwide, affecting individuals of all age groups [1]. It is particularly challenging for parents who have children diagnosed with food allergies, as they bear the responsibility of managing their child's condition and ensuring their safety [2]. The knowledge and awareness of parents, especially mothers, play a crucial role in preventing allergic reactions and providing appropriate care for their allergic children.

Previous research has shown that mothers, teachers, and other caregivers have a good understanding of food allergies. This has been reported in a number of studies. In 2018, a study was conducted in Jazan, Saudi Arabia to assess the level of knowledge among school teachers regarding food allergies in children. The findings revealed that the teachers possessed inadequate knowledge in this area, highlighting the pressing need for health awareness and education [3]. Another study carried out in 2015 found that urban school nurses have knowledge gaps and varied attitudes regarding food allergies [4], notably in relation to the treatment of reactions and perspectives of parents. As a consequence of these findings, primary healthcare practitioners and educational institutions have been tasked with developing and implementing an improved food allergy education and awareness program [5]. Nannies were found to have knowledge gaps on food allergies in children, according to a study that was carried out in 2014 [6]. However, a significant dearth of data persists about the quantification of food allergies' prevalence in Saudi Arabia. Significantly, a notable deficiency exists in the availability of comprehensive statistical data pertaining to the extent of maternal awareness regarding food allergies, despite the potential life-saving implications associated with possessing such knowledge. Consequently, the objective of this research was to assess the extent of knowledge and awareness pertaining to food allergies among mothers residing in the Aseer region of Saudi Arabia, whose children are afflicted with food allergies.

## Materials and methods

Study design

The present study utilized a cross-sectional research design to evaluate the level of knowledge and awareness pertaining to food allergies among mothers who have children with allergies in the Aseer region of Saudi Arabia. The process of data collection involved the administration of a standardized questionnaire to the participants.

Study participants

The participants for the study were recruited using a convenience sampling method. Mothers with allergic children in the Aseer region, Saudi Arabia, were selected as participants if they met the criteria of having at least one child with a medically diagnosed food allergy. A total of 400 mothers participated in the survey.

Data collection

The researchers utilized a structured questionnaire that they had prepared for the purpose of data gathering. The survey was a combination of multiple-choice and open-ended questions that were specifically formulated to evaluate the level of knowledge and consciousness of food allergies among mothers. The questionnaire was pilot-tested prior to the main data collection to ensure its clarity and appropriateness. The questionnaire covered several domains, including demographic information, knowledge and awareness about food allergies, the prevalence of allergenic foods, manifestations of food allergies, and factors influencing food allergies. The participants were asked to provide responses based on their understanding and experiences related to their allergic child.

Questionnaire validity measures

To assess the internal consistency reliability of the questionnaire used in this study, Cronbach's alpha was calculated. The Cronbach's alpha value is approximately 0.76, which indicates a moderate level of internal consistency among the knowledge questions.

Data analysis

The gathered data were inputted into a Microsoft Excel spreadsheet (Microsoft Corporation, Redmond, WA) and afterward imported into Python Jupyter Notebook (version 6.5.2) for the purpose of analysis. The researchers computed descriptive statistics, including frequencies and percentages, to provide a summary of the demographic features of the study participants, their levels of knowledge and awareness, the prevalence of allergenic foods, the symptoms of food allergies, and the factors that influence food allergies.

The study employed chi-squared tests to evaluate the relationships between several variables, including feeding techniques, the presence of other children with food allergies, and the incidence of food allergies. A p-value below 0.05 was deemed to be statistically significant.

## Results

Table 1 offers valuable insights into the demographics of mothers in terms of age, education, and occupation. On the age distribution of mothers in the given population, it reveals that there are 47 individuals between 18 and 30 years old, accounting for 25.27%, 65 mothers in the age range between 31 and 40 years, representing 34.95%, mothers aged 41 to 50 years old account for 36 individuals, making up 19.35%. The age group of 51 to 60 years comprises 14 mothers, accounting for 7.53% of the total population. Additionally, there are 12 mothers who are 60 years old and above, representing 6.45% of the total. The educational backgrounds of the mothers in the population reveal that among the mothers, 22 have completed primary school, making up 11.83%, 24 mothers have attained an intermediate school education, accounting for 12.9%, and 42 mothers have completed high school, representing 22.58% of the total population. The largest educational group consists of 79 mothers with a university education, accounting for 42.47%, and 19 mothers have pursued postgraduate education, making up 10.22% of the total population. Among the mothers, 72 are employed in governmental positions, accounting for 38.71% of the total. Additionally, 43 mothers work in the private sector, representing 23.12% of the total population. The category of housewives consists of 71 mothers who primarily focus on household duties, making up 38.17% of the total population.

**Table 1:** Sociodemographic characteristics of the participants

		N	%
Mother’s age	<18 years	12	6.45%
	From 18 to 30 years	47	25.27%
	From 31 to 40 years	65	34.95%
	From 41 to 50 years	36	19.35%
	From 51 to 60 years	14	7.53%
	From 60 years and above	12	6.45%
Mother's education	Primary school	22	11.83%
	Intermediate school	24	12.9%
	High school	42	22.58%
	University education	79	42.47%
	Postgraduate education	19	10.22%
Mother’s occupation	Governmental	72	38.7%
	Private	43	23.12%
	Housewife	71	38.17%
Mother’s monthly income	<5000 Saudi riyal (SR)	48	25.81%
	5000–10,000 SR	49	26.34%
	10,000–15,000 SR	50	26.88%
	15,000–20,000 SR	22	11.83%
	>20,000 SR	17	9.14%

It also shows that 25.81% of mothers (48) earn less than 5000 Saudi riyals (SR) each month. Additionally, 26.34% of mothers (49) earn 5000-10,000 SR. Additionally, 26.88% of mothers (50) earn 10,000-15,000 SR. Finally, 22 mothers (11.83% of the population) earn 15,000-20,000 SR.

The results in Table 2 provide insights into the knowledge and understanding of food allergies among the surveyed participants. In response to the question, "Do you think food allergies are transmitted to the baby through breastfeeding?", 54.84% of the participants correctly answered "No," with a frequency of 102. When asked about whether food allergies are considered a dangerous condition for their child, 65.59% of the participants responded with "Yes," totaling 122 individuals. Regarding the hereditary nature of food allergy, 51.08% of the participants recognized it as a hereditary condition, providing a frequency count of 95. In response to the question on whether a food allergy is an infectious condition, the majority of participants (68.82%) answered "No," indicating 128 individuals. When asked if food allergies are considered a chronic disease, 61.29% of the participants responded with "Yes," comprising a frequency count of 114. Lastly, when questioned about whether their child has an epinephrine pen and should carry it at all times, 59.68% of the participants responded with "Yes," with a frequency count of 111.

**Table 2:** Knowledge-based questions

Knowledge-based questions	Right answer	Frequency	Percentage (%)
Can food allergies be passed on to the baby through breastfeeding?	No	102	54.84
Do you believe food allergies pose a risk to your child's health?	Yes	122	65.59
Do you think food allergies are inherited?	Yes	95	51.08
Do you think food allergies are contagious?	No	128	68.82
Do you consider food allergies a chronic medical condition?	Yes	114	61.29
Does your child have an epinephrine pen that they should carry at all times?	Yes	111	59.68

Table 3 reveals the prevalence of food allergies associated with different food types among the surveyed participants, highlighting the significance of seafood and eggs as common allergens, as well as the presence of allergies to milk, wheat, chocolate, fruits, nuts, and other food types in the studied population. The most commonly reported food type associated with allergies is seafood (fish, shrimp, tuna), with a frequency of 74, representing 39.78% of the total responses. Eggs were the second most reported allergenic food, with a frequency of 42, accounting for 22.58% of the total responses. Milk allergies were reported by 12 participants, representing 6.45% of the responses. Other less frequently reported allergenic food types include wheat (five responses, 2.69%), chocolate (four responses, 2.15%), fruits such as banana and strawberry (nine responses, 4.84%), and nuts like peanut, hazelnut, and almond (six responses, 3.23%). Additionally, 34 participants reported other food types as allergens, representing 18.28% of the total responses.

**Table 3:** Food types causing allergy

Food type	Frequency	Percentage
Seafood (fish, shrimp, tuna)	74	39.78
Egg	42	22.58
Milk	12	6.45
Wheat	5	2.69
Chocolate	4	2.15
Fruits (banana, strawberry)	9	4.84
Nuts (peanut, hazelnut, almond)	6	3.23
Others	34	18.28

Table 4 provides information on the frequency and percentage of various manifestations associated with allergies. The skin manifestation table reveals that itching is the most common symptom reported by 50.54% of individuals, followed by dryness, pinpoint rash, raised circular rash, and eyelid and lip swelling. The respiratory manifestation table indicates that dyspnea is the most frequent symptom reported by 49.46% of individuals, while heaviness and wheezes are less common. The eye manifestation table shows that lacrimation (tearing) is the most reported symptom at 24.19%, followed by itching, redness, and eyelid swelling. The nasal manifestation table reveals that rhinorrhea (runny nose) is the most common symptom reported by 28.49% of individuals, followed by sneezing, congestion, and itching. Lastly, the gastrointestinal (GIT) manifestation table shows that abdominal pain is the most frequently reported symptom at 27.42%, followed by diarrhea, nausea, and vomiting. The other manifestations table highlights that fatigue and general tiredness are reported by 34.95% of individuals, while loss of consciousness is reported by 16.13%.

**Table 4:** Symptoms manifestation

Symptoms	Frequency	Percentage
Skin manifestation		
Itching	94	50.54
Dryness	30	16.13
Pinpoint rash	27	14.52
Raised circular rash	10	5.38
Eyelid and lips swelling	2	1.08
None	23	12.37
Respiratory manifestation		
Dyspnea	92	49.46
Heaviness	18	9.68
Wheezes	7	3.76
None	69	37.1
Eye manifestation		
Lacrimation	45	24.19
Itching	31	16.67
Redness	19	10.22
Eyelid swelling	16	8.6
None	75	40.32
Nasal manifestation		
Rhinorrhea	53	28.49
Sneezing	33	17.74
Congestion	18	9.68
Itching	16	8.6
None	66	35.48
Gastrointestinal manifestation		
Abdominal pain	51	27.42
Diarrhea	32	17.2
Nausea	21	11.29
Vomiting	9	4.84
None	73	39.25
Other manifestation		
Fatigue and general tiredness	65	34.95
Loss of consciousness	30	16.13
None	91	48.92

Table 5 presents data pertaining to several variables associated with food allergies and their corresponding connections. The table presents the frequency of children who have been medically diagnosed with food allergies, categorized by their respective feeding regimens. Based on the chi-squared test results, which yielded a p-value of 0.4925, it can be concluded that there is insufficient evidence to establish a meaningful relationship between the various feeding methods (i.e., both breastfeeding and artificial feeding) and the occurrence of food allergies as a medical diagnostic. The variable "Have other children with food allergy" represents the prevalence of individuals who have additional children affected by food allergies. The chi-squared test, with a p-value of 2.8e-18, suggests a statistically significant relationship between the presence of food allergies in a kid and the presence of food allergies in their siblings. The variable "Age of introducing solid food" represents the frequency at which solid food is introduced at various ages. Based on the chi-squared test results, which yielded a p-value of 0.6764, it can be concluded that there is insufficient evidence to support a significant link between the age at which solid food is introduced and the occurrence of food allergies.

**Table 5:** Relationship between child feeding, having other children with food allergies, age of introducing solid food, and being medically diagnosed

Variable		Medically diagnosed		Chi-squared test	P-value
		Yes	No		
Child feeding	Both	79	79	1.42	0.4925
	Artificial feeding	50	48		
	Breastfeeding	65	54		
Have other children with food allergy	Yes	164	71	76.02	2.8e-18
	No	104	220		
Age of introducing solid food	4 months	25	17	2.3238	0.6764
	6 months	43	49		
	8 months	50	53		
	1 year	36	31		
	After 1 year	32	31		

Table 6 offers significant insights about the various elements that may influence the occurrence of food allergies. This study aims to investigate the correlation between various feeding practices, including child feeding, artificial feeding, and breastfeeding, and the prevalence of medically confirmed food allergies. Based on the chi-squared test results, which yielded a p-value of 0.4925, it can be concluded that there is insufficient evidence to establish a meaningful relationship between the kind of feeding and the occurrence of food allergies as diagnosed medically. The study examines the relationship between the existence of food allergies in a current child and the presence of food allergies in other siblings. The statistical analysis, specifically the chi-squared test, demonstrates a strong and statistically significant link between having other children with food allergies and the development of food allergies in the current child. The statistical analysis conducted using the chi-squared test with a p-value of 0.6764 suggests that there is no statistically significant relationship between the age at which solid food is introduced and the occurrence of food allergies.

**Table 6:** Relationship between mother’s education, mother’s occupation, mother’s residence, and their knowledge

Variable		Knowledge		Chi-squared test	P-value
		Good	Poor		
Mother’s education	Primary school	17	27	26.077	3.0531E-05
	Intermediate school	17	29		
	High school	46	31		
	University education	110	51		
	Postgraduate education	16	23		
Mother’s occupation	Governmental	80	59	0.26499	0.8759
	Private	52	44		
	Housewife	74	58		
Mother’s residence	Urban	172	98	22.6422	1.9515E-06
	Rural	34	63		

## Discussion

The study's findings offer significant contributions to the understanding of food allergies, encompassing a range of topics such as demographic characteristics, knowledge and awareness levels, prevalence rates of allergenic foods, manifestations of allergies, and factors that contribute to the presence of food allergies. The age distribution exhibited a rather equitable distribution among various age cohorts, with the greatest percentage falling within the 31-40 years age bracket. In relation to the domain of education, a notable fraction of the mothers included in our study possessed a tertiary degree of education, so indicating a comparatively elevated educational attainment within the studied cohort. The predominant occupational category among mothers included in our study was governmental employment, followed by housewives and individuals engaged in the private sector. The findings of this study indicate that a significant proportion of mothers demonstrated accurate knowledge of important facets of food allergies, including the absence of allergen transmission during breastfeeding, the potentially severe consequences of food allergies, and the chronic nature of this disorder. The results of this investigation are consistent with the findings of a previous study conducted in Saudi Arabia by Alhuzimi and Alharbi [7], which also indicated a satisfactory degree of parental knowledge regarding food allergies. In our study, it was shown that seafood, specifically fish, prawns, and tuna, had the highest prevalence of reported allergic reactions. This finding aligns with the research conducted by Gupta et al. [8], which investigated the occurrence of prevalent dietary allergies. The review conducted by the authors identified seafood as a significant food group associated with allergies. In a similar vein, eggs emerged as the second most frequently reported allergenic food in our analysis. Eggs have constantly been recognized as a prevalent allergenic meal in numerous research conducted across various countries, including Saudi Arabia. In a study conducted by Alzahrani et al. [9], the researchers examined the incidence of food allergies in school-aged children in Saudi Arabia. The findings of the study indicated that eggs were frequently reported as one of the allergenic foods. Moreover, the present study revealed that milk, wheat, chocolate, fruits, nuts, and several other food categories were identified as extra-allergenic foods based on the responses provided by the subjects polled. The aforementioned findings are consistent with other scholarly investigations that have identified milk and wheat as prevalent allergies on a global scale. The prevalence of milk and wheat as allergenic foods in various communities has been revealed in a study conducted by Loh and Tang [10].

Skin manifestations, such as itching, dryness, pinpoint rash, raised circular rash, and eyelid and lip swelling, are often observed in clinical presentations in persons afflicted with food allergies. The results of this study align with prior research that has shown cutaneous symptoms as a prevalent manifestation of food allergies. In a study conducted by Lopez et al. [11], the clinical manifestations of food allergy were examined, revealing that common skin symptoms included itching, hives, and angioedema. Respiratory manifestations, including dyspnea (shortness of breath), heaviness, and wheezing, are frequently observed in conjunction with food allergies. This is consistent with the extensively documented respiratory symptoms observed in patients with food allergies. According to a study conducted by Gupta et al. [8], respiratory symptoms such as wheezing and shortness of breath were identified as prevalent manifestations of food allergies among adults in the United States. Eye manifestations, such as lacrimation (tearing), itching, redness, and eyelid swelling, are commonly observed in persons who suffer from food allergies. The aforementioned findings align with previous research that has established ocular symptoms as a manifestation of food allergies. In a scholarly investigation conducted by Elghoudi and Narchi [12], the eye manifestations of food allergy were examined, revealing the presence of symptoms such as pruritus, erythema, and edema of the eyelids. Symptoms often associated with food allergies include nasal manifestations, such as rhinorrhea (runny nose), sneezing, congestion, and itching. These findings are consistent with previous research studies that have identified nasal symptoms as a manifestation of food allergies. In a study conducted by Sicherer et al. [13], an examination of the clinical manifestations of food allergy revealed the presence of nasal symptoms, such as sneezing and congestion. Gastrointestinal (GIT) manifestations, such as abdominal pain, diarrhea, nausea, and vomiting, are commonly observed in patients who experience food allergies. The results of this study align with previous research that has highlighted gastrointestinal symptoms as a manifestation of food allergies. In a study conducted by Boyce et al. [14], an investigation was carried out on the clinical manifestations of food allergy. The findings of this study revealed the presence of gastrointestinal symptoms, such as abdominal discomfort and vomiting. In relation to the correlations among various feeding practices, including child eating, artificial feeding, and breastfeeding, and the prevalence of medically diagnosed food allergies, our research yielded results indicating a lack of statistically meaningful association. The aforementioned findings are consistent with prior research that has investigated the influence of feeding practices on the development of food allergies. The association between breastfeeding and the development of food allergies was investigated in a systematic study conducted by Greer et al. [15]. The review conducted by the authors determined that although breastfeeding offers a multitude of health advantages, it does not exhibit a substantial preventive impact on the occurrence of food allergies. Nevertheless, our research unveiled a statistically significant correlation between the presence of food allergies in other siblings and the prevalence of food allergies in the present offspring. The aforementioned findings align with prior research that emphasizes the impact of familial history on the emergence of food allergies. In a scholarly investigation conducted by Gupta et al. [8], the relationship between the presence of siblings with food allergies and the susceptibility of the present child to food allergies was examined. The study presented findings that indicate a noteworthy correlation, implying that familial factors contribute to the onset of food allergies. Regarding the timing of solid food introduction, our study did not identify a statistically significant correlation with the prevalence of food allergies. The aforementioned results are consistent with a systematic study conducted by Soares-Weiser et al. [16] about the optimal timing for introducing allergenic foods and their association with the development of food allergies. The review conducted by the authors presented a scarcity of evidence to substantiate a particular timeframe for the introduction of solid foods as a preventive measure against food allergies.

Nevertheless, it is imperative to recognize the limitations of the current investigation. The study population's sample size and demographic features may lack representativeness for the broader Saudi Arabian population or the entire Aseer region. Moreover, it should be noted that the cross-sectional design employed in this study imposes certain limitations on the establishment of causal correlations between variables. To enhance the validity and scope of the present study's findings, it is recommended that future research endeavors use larger sample numbers, longitudinal designs, and multi-center studies.

## Conclusions

This study provided valuable insights into the knowledge, awareness, prevalence, manifestations, and factors influencing food allergies among mothers with allergic children in the Aseer region, Saudi Arabia. The findings contribute to the existing body of knowledge on food allergies, highlighting the importance of targeted educational interventions, early identification of allergenic foods, and the influence of familial history on the development of food allergies. These findings can guide healthcare professionals, policymakers, and educational institutions in improving the management, prevention, and overall well-being of allergic children in the Aseer region and beyond.

## Appendices

Questionnaire

Section 1: Bio-Demographic Data

Mother's personal data

1. Mother’s age: - Less than 18 - 18-40 - More than 40

2. Nationality: - Saudi - Non-Saudi

3. Mother’s education: - Primary school - Intermediate school - High school - University education - Postgraduate education

4. Mother’s occupation: - Governmental - Private - Housewife - Other field

5. Residence: - Urban - Rural

6. Monthly income: - <5000 SR - 5000-10,000 SR - 10,000-15,000 SR - 15,000-20,000 SR - >20,000 SR

7. Family history of allergy: - None - Food allergy - Others

Section 2: Food Allergy Data for Children

1. Age of child in years: - <1 year - 1-4 - 5-9 - 10-14 - ≥15

2. Age at first allergy in years: - Before one year - 1-2 years - 3-6 years - Above 6 years

3. Gender of child: - Male - Female

4. Number of ER visits due to food allergy in the last 12 months: - None - 1-2 - >3

5. Have other children with food allergies: - Yes - No

6. Mechanism of food allergy: - Eating food - Smelling food - Touching food - Eating or smelling - All

7. Duration till signs of allergy: - Minutes to hours - Days

Section 3: Feeding Data of Children With a Food Allergy

1. Child feeding: - Breastfeeding - Formula - Both

2. Child had colostrum: - Yes - No

3. Age of first solid food: - 4 months - 6 months - 8 months - 1 year - After 1 year

4. Allergy after solid food intake: - Yes - No

Section 4: Knowledge of Food Allergy

1. Do you think food allergies are transmitted to the baby through breastfeeding? - Yes - No

2. Do you think that food allergy is a dangerous condition for your child? - Yes - No

3. Do you think that food allergy is a hereditary condition? - Yes - No

4. Do you think that food allergy is an infectious condition? - Yes - No

5. Do you think food allergy is a chronic disease? - Yes - No

6. Does your child have an epinephrine pen and should carry it at all times? - Yes - No

7. Knowledge about the most common types of food causing food allergies: - Seafood (fish, shrimp, tuna) - Egg - Milk - Wheat - Chocolate - Fruits (banana, strawberry) - Nuts (peanut, hazelnut, almond) - Others

8. Knowledge about the symptoms of food allergy as recorded for children by their mothers:

Skin manifestations: - None - Itching - Dryness - Pinpoint rash - Raised circular rash - Eyelid and lips swelling - Others

Respiratory manifestations: - None - Dyspnea - Heaviness - Wheezes - Cough

Eye manifestations: - None - Lacrimation - Itching - Redness - Eyelid swelling

Nasal manifestations: - None - Rhinorrhea - Sneezing - Congestion - Itching - Others

Gastrointestinal (GIT) manifestations: - None - Abdominal pain - Diarrhea - Nausea and vomiting

Others: - Fatigue and general exhaustion - None

9. Medically diagnosed: - Yes - No
